# Duty hour restrictions: organizational dynamics, systems issues, and the impact on faculty

**DOI:** 10.1186/1472-6920-14-S1-S5

**Published:** 2014-12-11

**Authors:** Glen Bandiera, Melissa Kennedy Hynes, Salvatore M Spadafora

**Affiliations:** 1 University of Toronto, Toronto, Ontario, Canada; 2 St. Michael’s Hospital, Toronto, Ontario, Canada; 3 Mount Sinai Hospital, Toronto, Ontario, Canada

## Abstract

The potential impact of resident duty hour restrictions on faculty is likely significant; however, the extent of this impact has still not been well documented. We undertook a narrative review of the literature to determine the magnitude of that potential impact and the nature of the evolving discourse related to faculty members as individuals. The literature provides an inconsistent picture of the impact of duty hour restrictions on faculty. While some studies have reported a significant increase in faculty workload, others suggest that the impact of duty hour restrictions has been minimal. Some papers suggest that duty hour restrictions may fundamentally change the nature of resident–teacher interactions and, as a result, will necessitate significant changes to the way education is delivered. Overall, the majority of issues of concern relate to one of the following: volume and composition of work, impact on faculty career choice, evolving perceptions of residents as learners, and the need to find an appropriate balance between learning and the quality and quantity of patient care. In describing these themes we identify some potential solutions and future directions for reconciling duty hour restrictions with faculty perceptions, anxieties, and desired outcomes.

## Introduction

The body of literature on resident duty hour restrictions has neither systematically nor extensively dealt with the potential impact of these restrictions on faculty. The 1984 death of Libby Zion is remembered by many as the impetus for the 2003 US duty hour restrictions, but it also brought about a new imperative for faculty to provide closer and more attentive supervision of residents [1]. However, what impact would that increased supervision have on faculty? The 2010 National Physician Survey in Canada confirmed that physicians in practice are working long hours [2]. In this survey, practising specialists reported working a mean of 53 hours per week exclusive of call. Call coverage added an average of 25 hours per week, and this often included 24**-**hour continuous coverage. In addition, the majority of practising specialists reported the need to continue professional duties immediately following the completion of long shifts. As well, many teaching faculties have modelled their professional activities, including clinical service, administration, research, and education with the expectation that residents will contribute to a certain degree to patient care within a supervised academic environment.

The evidence in favour of duty hour restrictions has, in some cases, led to changes without consideration of, or investigation into, the implications for all participants in the health care system, including faculty. Restrictions to the duty hours of residents will challenge the manner in which faculty work, the way they teach, and the view they have of the future of medical education. We sought to survey the literature on the effects of duty hour restrictions on faculty members as individuals and to summarize the key thematic findings in this paper.

## Methods

A comprehensive search of PubMed and Ovid was performed using key words such as “duty hours” or “resident work hours” and “faculty” and/or “teachers” and/or “Program Directors” and/or “impact” and/or “system” and/or “postgraduate” to capture information relevant to the impact of duty hour restrictions on teaching faculty. The review was restricted to studies that explicitly addressed, as a central subject, duty hours with respect to their perception by or impact on faculty or teachers or the associated impact on faculty as related to the other topic areas.

Separate searches of Ovid Embase (1974 to present) and PubMed (1980 to present) yielded a total of 152 abstracts. These abstracts included both qualitative and quantitative research published in English (although not necessarily specific to the Canadian experience). As well, because the number of articles pertaining to the impact of duty hour restrictions on faculty is relatively small, all types of published articles – including empirical research, editorials, comments, and opinions – were included in the review.

The PubMed search yielded 81 articles, for which all abstracts were reviewed and examined for duplication across the Ovid database search. In addition, 32 additional abstracts were reviewed from a search conducted by librarians at the Royal College of Physicians and Surgeons of Canada. Along with the database searches, grey literature from the websites of government and other relevant organizations that deal with duty hour regulations in Canada and the United States was searched.

In total, 40 full-text articles were retained from the first review and were then screened by at least two of the authors. A final list of 24 published, refereed journal articles and/or grey literature was generated.

Rather than define a priori areas of focus, we elected to develop a series of key themes that arose through review of the published literature and to categorize our findings accordingly.

## Results

There is some discordance in the literature on the effects of duty hour restrictions on faculty. This is due in large part to a lack of systematic enquiry and to the fact that few attempts have been made to validate observations that have arisen through open-ended, observational, or exploratory research. For example, while some studies have reported a significant increase in workload for faculty and identified their evolving perception of the learner, other studies have suggested that duty hour restrictions on faculty have had a minimal impact since their adoption in Canada [3-7]. Nevertheless, the following themes were identified: volume and composition of work, impact on faculty career choice, evolving perceptions of residents as learners, and the need to find an appropriate balance between learning and the quality and quantity of patient care.

### General impact of resident duty hour restrictions on faculty

The combined effects of restricted resident duty hours, the changing roles of staff physicians, variable responses from teaching hospitals to develop alternative financially feasible models of care, and the unrelenting need to maintain – and, in many cases, increase – service provision has created a precarious situation for individual faculty members. Hypothesized implications include increased faculty workload, the need for faculty to remain positive professional role models for residents during a time of perceived identity crisis (among the many who believe that always being available for patients is a defining characteristic of physicians), and the mandate to ensure adequacy of trainee supervision [6-10].

Since the restriction in duty hours reduces the service contribution previously made by residents and relied upon by teaching hospitals, these institutions are also developing solutions that ensure service levels are maintained within existing budgetary constraints.

### Volume and composition of work

Winslow and colleagues examined faculty hours in surgical subspecialties when duty hour restrictions were introduced in the United States in 2003. Prior to the introduction of duty hour restrictions, 70% of faculty thought that their work hours would increase [11]. Five years later, Goitein and colleagues reported that, since the adoption of duty hour restrictions, faculty spent more time on clinical work (52%), felt more responsibility for supervising patient care (65%), and spent less time on research (51%) and resident education (72%) [5]. Similarly, Vanderveenin found that 56% of faculty felt they had less time for teaching since the adoption of reduced duty hours [4]. Learners have validated faculty perceptions in this regard [12,13]. In addition, Arnold and colleagues studied medical students’ perceptions of a career in surgery both before and after the introduction of duty hour restrictions. Although students’ perceptions of a surgeon’s lifestyle improved significantly after the duty hour restrictions, students showed no increased interest in surgery as a career [14].

The selective reduction in work hours realized by directing some duties to physician assistants, nurse practitioners, or other provider groups is met with caution by some faculty. They indicated that it is important for residents to learn how to do this type of work in order to become well-rounded physicians [6].

### Impact on faculty career choice

Girard and colleagues found that faculty career satisfaction was closely associated with both educational experiences and strong interpersonal interactions [15]. Furthermore, faculty who were engaged in teaching and in interacting with students and colleagues tended to be more satisfied with their jobs. The National Physician Survey reported that the most important factor influencing whether a physician has a satisfying and successful medical practice is the ability to achieve a balance between work life and personal life [2]. This finding aligns with the work of Leduc and colleagues, who found that workload and lifestyle are two major factors in career choice and practice location [16].

Goiten and colleagues completed a study on the effects of resident work hour restrictions on the professional lives of faculty and reported that changes in teaching time (or lack thereof) was independently associated with the probability of faculty leaving academic medicine within the next three years. While Goiten’s prediction is just that, it is something that medical educators must begin to take into consideration [5]. Traditionally, physicians who decided to go into a teaching versus a non-teaching practice did so because of their desire to teach, but also because teaching afforded some benefit with reduced clinical work hours, often at the cost of an associated reduction in income. As workload issues gain an increased foothold in the discussion of faculty and duty hour restrictions it is possible that physicians will choose non-teaching practices in order to achieve satisfaction by having more time available to take part in activities outside the workplace [15,16].

### Evolving perceptions of residents as learners

In 2006, Coverdill reported that “duty-hour restrictions represented a disruption to the symbolic aspects of a traditional status hierarchy” [3]. Faculty felt that duty hour restrictions not only increased their workload but also required faculty to do clinical work that would traditionally have been done by residents.

Schuster concluded that faculty felt duty hour restrictions were associated with a “decrease in didactic teaching, bedside teaching, resident execution of procedures, resident autonomy, and overall resident education [17].” This study also revealed that residents were less accountable to their patients after the introduction of restricted duty hours. Faculty felt that this was not best practice and was a shift from how they themselves had learned. Faculty also felt that residents under the duty hour restrictions were less accountable for patient care and did not develop good physician–patient relationships when compared with residents in years past.

While the practice of open dialogue – where the learner is an active participant in teaching and learning – is widely encouraged in residency training today, it is also essential that faculty guide residents as they move toward greater independence [18]. As well, the use of open dialogue can change the way learners and faculty interact and perceive each other, both inside and outside the classroom. With the coincident advent of duty hour restrictions, it is possible that the shift toward an open dialogue model has exacerbated tension between the “way things were done” versus “the way they are done now.”

Faculty struggle with exemplifying and teaching professionalism in the wake of duty hour restrictions, and some authors identify this challenge as a call to action for educators [10]. Duty hour restrictions have certainly created difficulties for some faculty as they attempt to teach while, at the same time, respecting the restrictions [19]. An even greater challenge is the creation of curricula that embrace “the development of professional values, actions, and aspirations” and maintain professionalism as “the backbone of medical education [20].” This notion of how professionalism is perceived is linked to the traditional model of learning in the postgraduate environment. Woodrow and colleagues are blunt when they suggest that “instead of trying to adapt the traditional model of apprenticeship training to fit into these emerging shorter duty hours, a new structure of postgraduate training grounded in the principles of educational theory, incorporating evidence-based educational strategies, and tailored to evolving health care environments, may be required [19].”

### Finding a balance between learning and quality and quantity of care

Jamal and colleagues conducted a systematic review of the effects of the Accreditation Council for Graduate Medical Education (ACGME) duty hour restrictions on surgical disciplines [21]. Faculty opinions indicated widespread concerns about patient care and, specifically, continuity of care. This perception appears to have some justification. In 2004, Chung and colleagues reported that, following the introduction of duty hour restrictions, residents performed fewer operations, saw fewer patients, and attended fewer conferences [22]. Although this is primarily a problem for residents, it is also troubling for faculty who strive to both ensure continuity of excellent care for patients and to maximize hands-on experience for future independent practitioners. While most of these findings represent faculty opinion rather than measured effects on care, the feeling that one is not providing optimum care to patients and experiences for learners is sure to have a negative effect on faculty members’ self-worth and work satisfaction.

Bismilla and colleagues, looking at the effects of a reduction in duty hours from 28 to 24 in Ontario, demonstrated that a decrease in direct staff supervision was seen more often at the junior resident level than at the senior resident level. Given that staff supervision is a critical element in patient safety, the potential impact of reduced direct supervision will need to be studied [23].

Concern exists that there may not be enough time for teaching itself. Dimitris and colleagues implied that increased workload and a reduced number of residents in the hospital inevitably result in less time for teaching. The combination of reduced hours spent in the hospital and the potential workload increase for faculty results in less faculty time to provide experience in the operating room and to teach in clinics. Dimitris cautioned this could lead to decreased competency [7].

While duty hour restrictions have been implemented for all providers, including faculty, in some jurisdictions (e.g., France), we found little literature related to faculty perceptions about restrictions on their own duty hours. Most jurisdictions leave the issue of physician work hours to the oversight of individual self-regulating professionals. It seems that restricting duty hours for faculty has thus far drawn less attention than it has for residents. Issues around equity among all caregivers, autonomy for professionals, and the additional impediments to providing care that might be associated with faculty work hour restrictions were not adequately addressed in the literature.

## Discussion

Until recently there has been fear, hypothetical prognosticating, and reactionary resistance, along with justifiable concern, around duty hour restrictions and their effect on faculty. Our categorization of the varied issues in the literature now provides some basis for informed discussion. We first provide some general commentary on our findings and then address considerations for change and adaptation, with a focus on faculty members as individuals.

### The impact of resident duty hour restrictions on faculty

There is concern that reduced resident work hours will translate directly into more work that will need to be done by others, and it is easy to assume that faculty will make up the deficit. This, of course, presumes that the volume, scope, and model of care provision remain the same. Judging from a number of studies referenced above, faculty have felt this pressure both directly and indirectly. Evidence suggests faculty are indeed working more following the implementation of resident duty hour restrictions. Faculty worry about the impact the reduction in resident work hours has on patient care, educational quality, and the eventual competence of graduating residents. Several questions arise, as follows:

• Can we afford to pay faculty to do work previously done by residents?

• Are the hypothetical costs of less research, compromised educational rigour, and reduced capacity for innovation “worth it”?

• With the perceived traditional tangible benefits of academic practice being challenged, will academic work become too much of a burden in the wake of duty hour restrictions?

In addition, a related issue is the dissonance between what is expected of residents and the career experience of faculty. This is especially relevant if faculty members are expected to fill the gaps in care provision created by resident duty hour restrictions. Recent graduates could find themselves in a position where they are expected to provide a greater level of service and to work longer hours than they were accustomed to as residents. While evidence suggests that faculty members derive intrinsic rewards from academic practice, concern over future work–life balance for current residents is pervasive. To address these concerns, evolution in models of care and education, as well as perceptions of the role of physicians, will need to be examined going forward.

The emergence of duty hour restrictions has brought about changes that some faculty see as a step backward in establishing the professional identity that is required of a dedicated physician. Open dialogue between faculty and learners will be required if they are to develop a definition of professionalism that is not based on the number of hours worked, but rather on physicians’ skills and how physicians are viewed by their peers and patients. The emergence of duty hour restrictions has been viewed by some faculty as contradictory to the way professionalism in medicine should be defined and modelled. The ability of individual faculty members to provide adequate time for dialogue and discussion will affect how residents communicate with each other, with staff, and, ultimately, with patients. Providing opportunities for discourse between faculty and learners through different learning environments is pivotal to allowing residents to maintain an open dialogue and manage their professional conversations [19].

Faculty are somewhat concerned – and are, at times, anxious – that less hands-on, experiential learning time and a reduced hierarchy may further decrease the autonomy of graduates that is required by the accrediting colleges. Establishing confidence among faculty in the new models of residency training and ensuring that they themselves will not bear the burden of an increased clinical workload will be critical to enabling faculty members to reorient their expectations of professional practice so that duty hour restrictions are viewed as positive, adaptive, and protective advancements.

### Impact on the system: possible solutions for change

Many hospitals have hired alternative providers and physician extenders (e.g., physician assistants, nurse practitioners, clinical associates) to compensate for resident duty hour restrictions. The use of physician extenders is appealing because hiring more residents to do the work may not be feasible and, without an eventual job market for graduates, could be seen as somewhat irresponsible and as a poor use of educational resources. Although physician assistants have reportedly reduced some of the work that physicians found themselves doing in the absence of residents, their salaries are a financial burden on hospitals. As well, with the use of these physician extenders, some teaching faculty may worry that resident learning opportunities will disappear.

There is an inherent conflict between keeping the hospital functioning at night while at the same time adhering to teaching methods that concentrate activities in the typical working days within the traditional teaching and patient care systems. New resident work hour models may reduce the overlapping time that both faculty and residents are present in the practice environment to mutually engage in teaching activities.

## Handover and how it affects faculty

While duty hour restrictions were introduced in an attempt to increase patient safety, an increase in the number of handovers performed by residents who are not adequately trained may be having the opposite effect [24]. Faculty members will need to develop confidence in handover models and will also need to teach and oversee the care delivered by a changing team of residents. Advancing this will require teaching time and human resources from an already stretched teaching faculty. Potential solutions include giving senior residents more autonomy and responsibility for teaching junior residents the skills necessary for effective patient handover [24] and including safety competencies in the curriculum [25,26].

## Conclusion

The literature to date consists primarily of faculty’s subjective opinions of the impact of changes associated with duty hour restrictions. It remains unclear how significant the effects of reduced duty hours will be, but anxieties exist nonetheless. There are also tensions related to the role of faculty in the wake of duty hour restrictions. The major themes are volume and composition of work, impact on faculty career choice, evolving perceptions of residents as learners, and the need to find a balance between learning and patient care.

A thoughtful, engaged, and dedicated academic faculty will be crucial to address the many changes and challenges associated with duty hour restrictions. As well, faculty development and dialogue will be critical in the process of planning and implementing resident duty hour restrictions. Moving forward, academic institutions, teaching hospitals, and residency programs must continue to look for ways to optimize experiences for faculty within the confines of the duty hour restrictions.

## Authors’ contributions

The authors contributed equally to this work. MKH participated in the literature search and the data review and synthesis with the assistance of GB and SMS. GB and SMS led the development of the manuscript. All participated in the data analysis and the writing and revision of the final paper. All authors read and approved the final manuscript.

## Competing interests

The authors have none to declare.

## Declarations

Resources and secretariat support for this project was provided by the Royal College.

This article has been published as part of *BMC Medical Education* Volume 14 Supplement 1, 2014: Resident duty hours across borders: an international perspective. The full contents of the supplement are available online at http://www.biomedcentral.com/bmcmededuc/supplements/14/S1. This article was submitted in 2011 and peer reviewed in 2012. Final acceptance for publication as part of this supplement was in 2014.

Publication of this supplement was supported and funded by the Royal College of Physicians and Surgeons of Canada. The funding agency played no role in the design, in the collection, analysis and interpretation of data; in the writing of the manuscripts; and in the decision to submit the manuscript for publication. The articles have been through the journal's standard peer review process for supplements. The Supplement Editors declare that they have no competing interests.
